# Mid-infrared photon sensing using InGaN/GaN nanodisks via intersubband absorption

**DOI:** 10.1038/s41598-022-08323-9

**Published:** 2022-03-11

**Authors:** Zhang Xing, Afroja Akter, Hyun S. Kum, Yongmin Baek, Yong-Ho Ra, Geonwook Yoo, Kyusang Lee, Zetian Mi, Junseok Heo

**Affiliations:** 1 Semiconductor Industry and Technology Research Institute, Jimei University, Xiamen, 361021 China; 2 Department of Electrical and Computer Engineering, Ajou University, Suwon, 16499 South Korea; 3 Department of Electrical and Electronic Engineering, Yonsei University, Seoul, 03722 South Korea; 4 Department of Electrical and Computer Engineering, University of Virginia, Charlottesville, VA 22904 USA; 5 Optic and Electronic Component Material Center, Korea Institute of Ceramic Engineering and Technology, Jinju, 52851 South Korea; 6 School of Electronic Engineering, Soongsil University, Seoul, 06978 South Korea; 7 Department of Electrical Engineering and Computer Science, University of Michigan, Ann Arbor, MI 48109 USA

**Keywords:** Electronic properties and materials, Nanowires

## Abstract

Intersubband (intraband) transitions allow absorption of photons in the infrared spectral regime, which is essential for IR-photodetector and optical communication applications. Among various technologies, nanodisks embedded in nanowires offer a unique opportunity to be utilized in intraband devices due to the ease of tuning the fundamental parameters such as strain distribution, band energy, and confinement of the active region. Here, we show the transverse electric polarized intraband absorption using InGaN/GaN nanodisks cladded by AlGaN. Fourier transform infrared reflection (FTIR) measurement confirms absorption of normal incident in-plane transverse electric polarized photons in the mid-IR regime (wavelength of ~ 15 μm) at room temperature. The momentum matrix of the nanodisk energy states indicates electron transition from the ground state *s* into the *p*_*x*_ or *p*_*y*_ orbital-like excited states. Furthermore, the absorption characteristics depending on the indium composition and nanowire diameter exhibits tunability of the intraband absorption spectra within the nanodisks. We believe nanodisks embedded nanowires is a promising technology for achieving tunable detection of photons in the IR spectrum.

## Introduction

Intersubband (Intraband) absorption in semiconducting materials has recently attracted interest due to its intrinsic advantages for their applications in spectroscopy, photodetection, and terabit optical communications. By utilizing intraband transitions of electrons with high mobility, devices such as quantum cascade lasers and infrared (IR) photodetectors can exhibit superior efficiency, narrow line width, fast modulation speed, and large output powers at room temperature^1–8^. Furthermore, the transition probability of electrons within the conduction band is much higher than interband transitions, which involves both the valence and conduction bands^9–12^. Recently, the intraband absorption has been confirmed in the near- to mid-infrared spectrum using GaN/AlGaN multiple quantum well (MQWs)^12–18^, coupled double quantum well^19^, and GaN/AlN quantum dot (QD)^20–23^ material systems. However, intraband absorption of transverse electric (TE) polarized light can be hardly achieved using quantum well based structures; a consequence of intraband selection rules. This imposes limitations for application of quantum well systems for intraband transition since normal incidence absorption is forbidden. However, this limitation can be eliminated using quantum wire, disk, and dot structures alternative to the planar quantum well devices. Moreover, the nanowire-based IR photodetectors show a lower dark current compared to that of IR photodetectors based on planar quantum wells^24^. The high aspect ratio and surface-to-volume ratio of nanowire heterostructures allow epitaxial growth of large lattice mismatch material systems with minimal defects compared to the identical planar heterostructures, leading to superior quantum efficiency^25–35^. In this work, we present theoretical and experimental evidence of intraband absorption in InGaN/GaN nanodisks cladded by AlGaN. First, the normal incidence intraband absorption in the quantum disk embedded nanowires is theoretically investigated by solving the single band Schrödinger equation and taking into consideration the strain effect presented in this heterostructure. Then, the absorption of normal incident TE polarized light in III-nitride nanowire heterostructures is experimentally demonstrated. The absorption energies are experimentally verified by measuring the absorption spectrum of semi-freestanding nanowires transferred on a thin CYTOP film. Fourier transform infrared spectroscopy (FTIR) of the nanowires show intraband absorption at room temperature in the mid-IR regime at a wavelength of ~ 15 µm (82 meV). Furthermore, the intraband absorption energy as a function of indium composition and the radius of the nanowire indicates that higher indium concentration induces a stronger internal electrical field, leading to a deeper potential in the nanodisk, which consequently leads to a blue-shift in the absorption energy, whereas an increase in nanowire radius reduce the absorption energy due to a relaxed quantum confinement.

## Results and discussion

**Figure 1:**
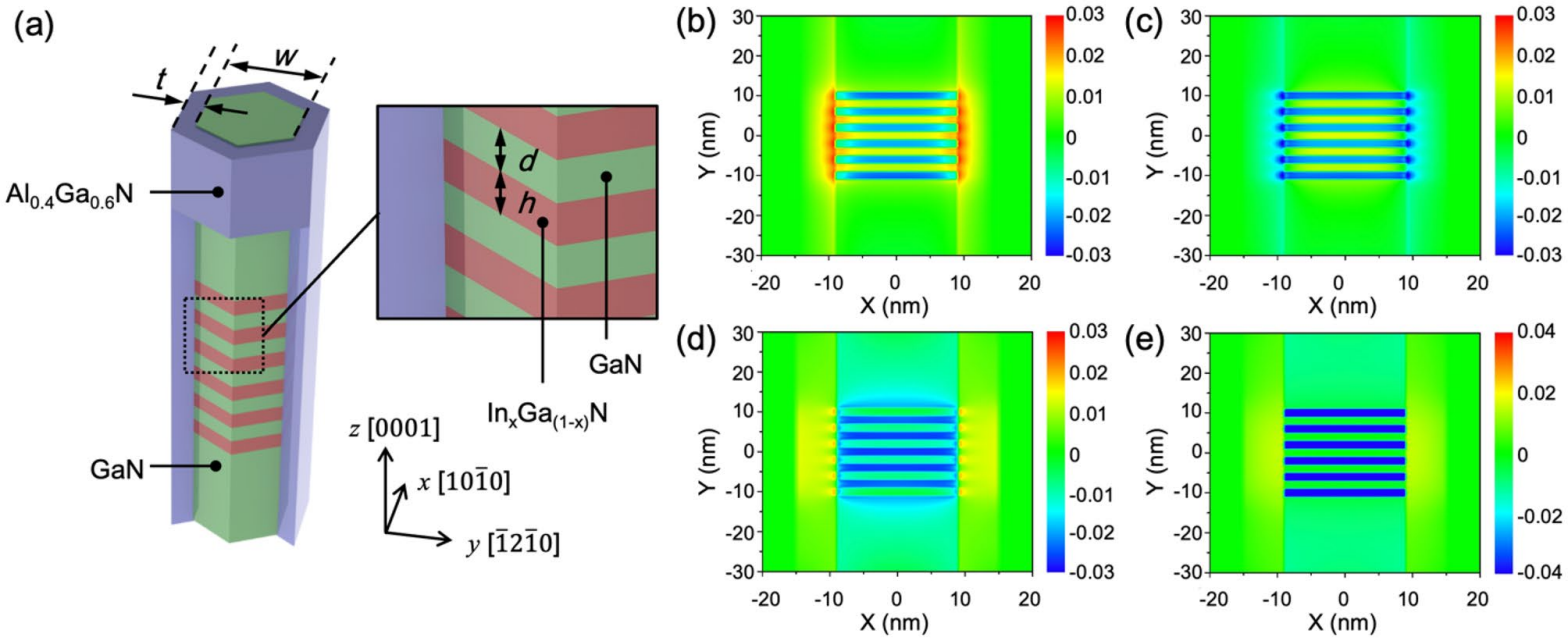
(**a**) Schematic view of the InGaN disk-in-nanowire embedded within a GaN/AlGaN core–shell structure. Strain components of (**b**) *e_xx*, (**c**) *e_yy*, and (**d**) *e_zz* in the x–y plane. (**e**) Hydrostatic strain in the InGaN/GaN nanodisk surrounded by an Al_0.4_Ga_0.6_N shell (30% In, *w* = 20 nm, *t* = 2 nm, *d* = 2 nm).

A schematic illustration of the simulated nanowire heterostructure is shown in Fig. 1a, indicating the relevant dimensions and orientation of the nanodisk embedded nanowire. The diameter (*w*) of the nanowire core is approximately 20 nm surrounded by a 5 nm thick Al_0.4_Ga_0.6_N cladding (*t*) which acts as a strain-compensator^36–40^. The In_x_Ga_1−x_N nanodisk is 2 nm thick (*h*) and separated by a 2 nm GaN barrier (*d*). The top and bottom of the nanowire is capped by 30 nm thick GaN. First, we have modeled the two-dimensional strain distribution of the core–shell nanowire using the Nextnano3™ simulation package^41^ by considering a strain minimized model with Neumann boundary conditions (i.e*.* no external forces acting on the sample and the derivative of the stress tensors is zero at the boundaries). The calculation is based on a continuum mechanical model from classical elasticity (see Supplementary Information). Figure 1b–e shows the cross-sectional view of the strain components of the core–shell nanowire with an indium composition of 30%. The InGaN nanodisk is under compressive strain (negative values represent compressive strain and positive values represent tensile strain) in both *e*_*xx*_ and *e*_*yy*_, and relaxed in the *e*_*zz*_ due to the lattice mismatch between InGaN and GaN. The GaN barriers, on the other hand, are relaxed in the *e*_*xx*_ and *e*_*yy*_ and compressively strained in the *e*_*zz*_, resulting in a net tensile strain at the interface between the InGaN/GaN nanodisk pairs and the AlGaN shell. Consequently, the hydrostatic strain of the InGaN nanodisks is compressively strained near the center of the nanowire, but tensile near the GaN/AlGaN core–shell interface. This allows formation of a potential well for efficient carrier accumulation in the nanodisks.

**Figure 2:**
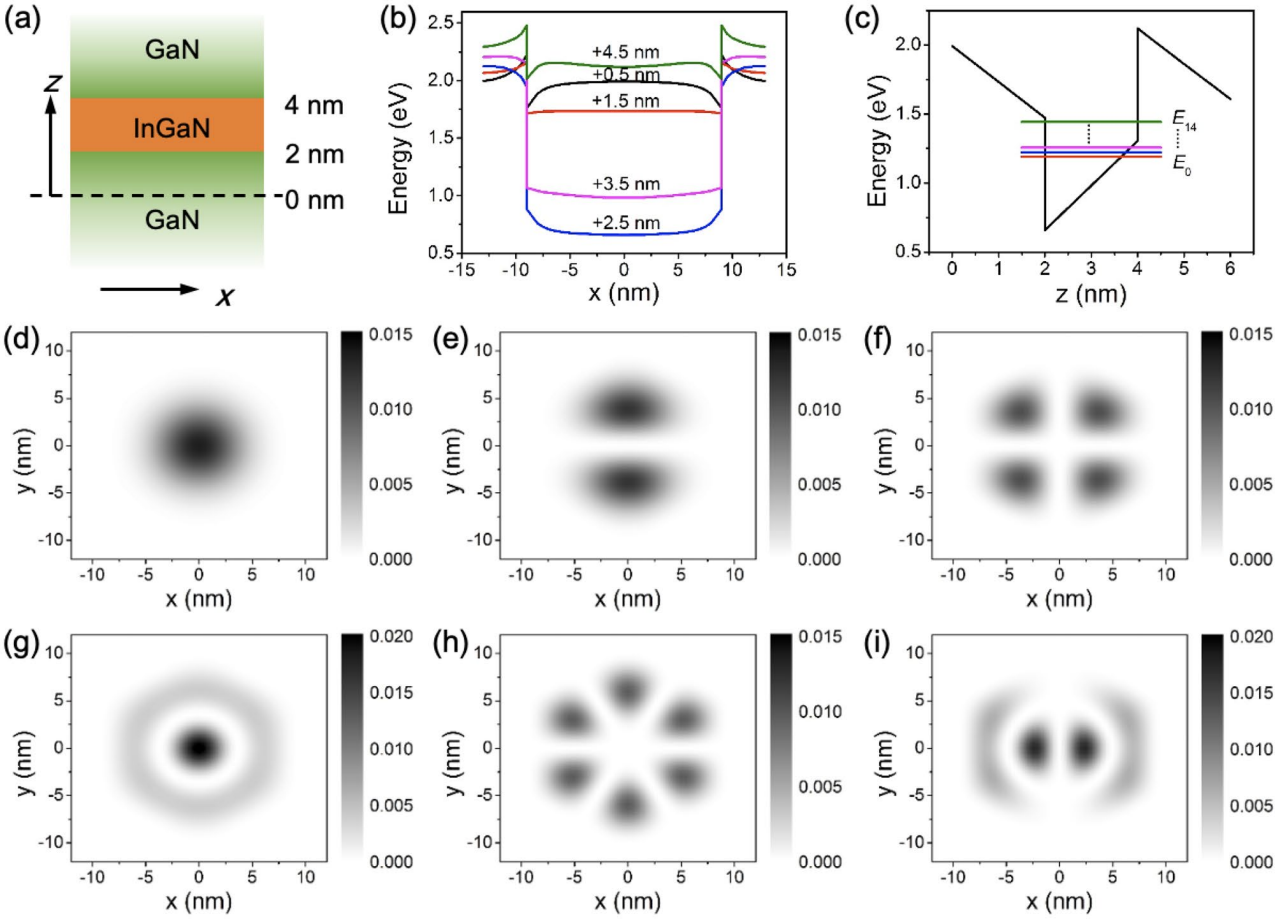
(**a**) Cross sectional schematic of the nanowire core in the growth direction. Conduction band energy profiles (**b**) in-plane and (**c**) along the *z*-axis. Confined energy levels are also shown in (**c**). Probability density functions of electrons for the first 6 non-degenerate states in the InGaN nanodisk. (**d**) Ground (*E*_0_) state, (**e**) *E*_1_ state (*p*_*y*_ orbital-like) which is degenerate with the *E*_2_ state (*p*_*x*_ orbital-like not shown here), (**f**)–(**i**) *E*_3,_ *E*_5_, *E*_6_, *E*_8_ states.

The simulated conduction band energy profile, considering the strain induced piezoelectric field in the in-plane (*x*-direction) and growth direction (*z*-direction) is shown in Fig. 2a–c. Using the coordinates defined in Fig. 2a, the in-plane conduction band profile along the length of the nanowire is shown in Fig. 2b. Due to the potential well formed by strain, energetically favorable regions for electron accumulation shift from the outer edges of the nanowire (the interface between the InGaN/GaN core and AlGaN shell) to the center of the InGaN disk. The presence of a spontaneous piezoelectric field in the InGaN layer is due to the highly strained polar InGaN/GaN interface (shown in Fig. 2c).

**Figure 3:**
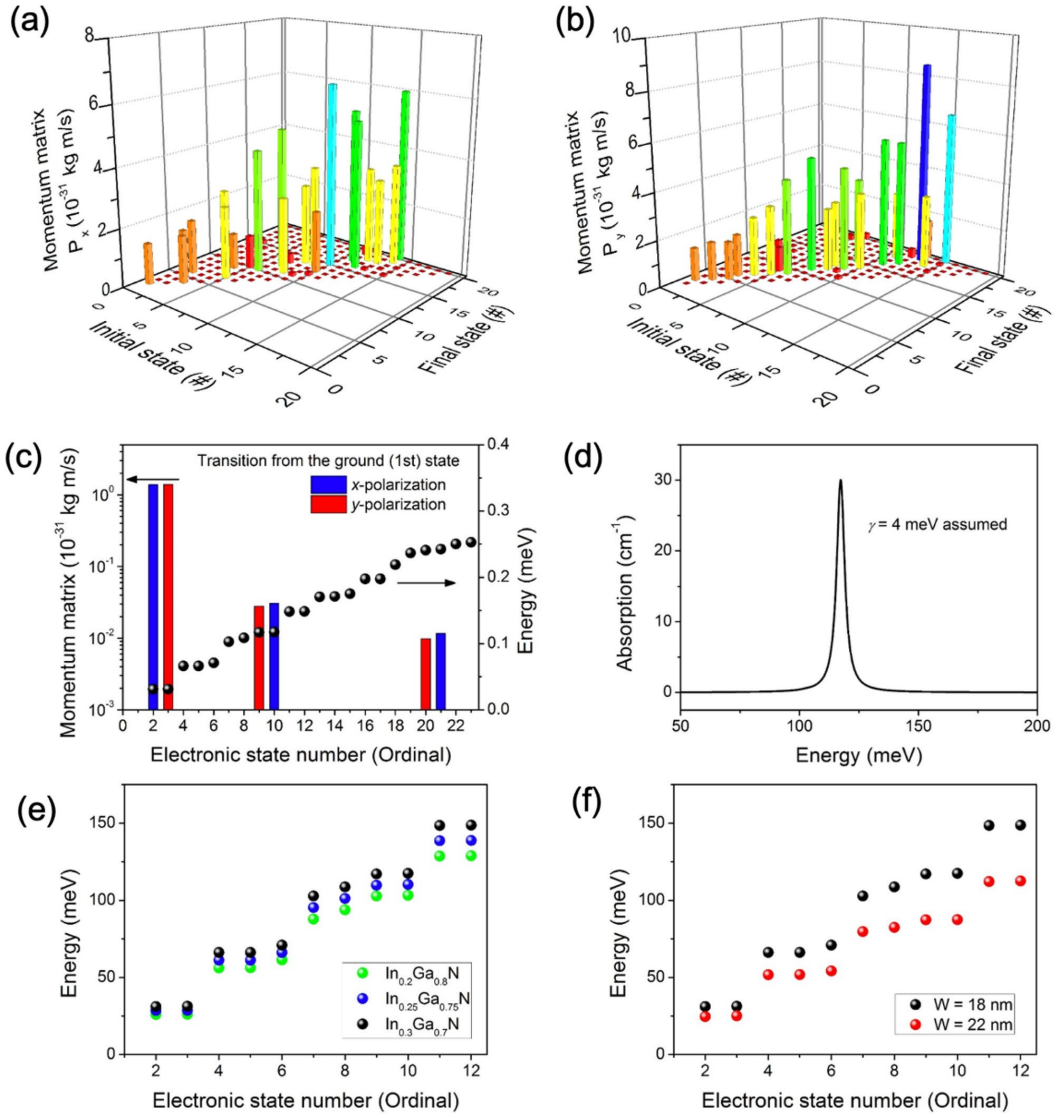
Momentum matrix element of transitions from various initial states to higher excited states by (**a**) *x*-polarized and (**b**) *y*-polarized light. (**c**) In-plane momentum matrix element and energy distribution for ground state to higher excited state transition, (**d**) intraband absorption with 4 meV FWHM for a In_0.3_Ga_0.7_N nanodisk with a diameter of 20 nm. Intraband absorption energy distribution for various (**e**) indium composition and (**f**) diameter of the nanodisk.

Figure 2d–i show cross-sectional electron probability density functions of the first 6 non-degenerate states in the In_0.3_Ga_0.7_N disks (1st, 2nd, 4th, 6th, 7th, and 9th states), calculated using single-band Schrödinger equation with Dirichlet boundary conditions. Figure 3a,b show a map of the momentum matrix elements for *x*- and *y*-polarized light within the nanodisk, indicating transition probabilities for electrons in an arbitrary energy state to a higher energy excited state. At room temperature, the ground state is fully occupied while the excited states are empty. Therefore, it is beneficial to take a closer look at the transition dynamics of electrons from the initial (1st) ground state to higher excited states. The momentum matrix and energies representing transitions from the ground states to higher ordinal states are shown in Fig. 3c. We note that the momentum matrix generally follows the transition rate obtained by invoking Fermi’s golden rule (see Supplementary Information). The transitions from the ground state into excited states that are similar to *p*_*x*_ or *p*_*y*_ orbitals can be observed, which is an indication of TE polarized photon absorption. Figure 3d shows a simulated mid-IR intraband absorption from the ground state to the 9th and 10th excited states, assuming a full width at half maximum (FWHM) of 4 meV, while the lower energy level absorptions due to the thermal energy at room temperature are not significant. A higher doping concentration and photoexcitation in the nanodisks than what is used in the simulation may potentially induce free-carrier screening effects^32,42^.

It is generally known that the absorption intensity and energy can be tuned by strain, confinement area, shape, and composition of the quantum confined structure (in our case, the nanodisk). Hence, we have investigated the tunability of the intraband absorption in nanodisk structures. Figure 3e shows the intraband absorption energy as a function of indium composition values of 20%, 25%, and 30%. As mentioned previously, the excitation from the ground state to the first two excited states for all indium composition is negligible due to the thermal energy at room temperature. A linear correlation between the indium composition and the absorption energy was predicted; the absorption energy increases as the indium composition increase. Increased indium content leads to a higher compressive strain, leading to higher built-in piezoelectric field within the nanodisk which induces a sharper confinement potential and larger energy gaps between the ground state and excited states. Additionally, higher indium concentration lowers the ground state energy towards the Fermi level. Consequently, the intraband absorption energy increases, i.e. blue-shifted. Similarly, the effect of the nanodisk diameter on the intraband absorption energy is shown in Fig. 3f. As the nanodisk diameter decreases from 22 to 18 nm, a significant blue-shift occurs due to the stronger confinement within the InGaN nanodisk. The diameter of the nanowire has a more significant impact on the absorption energy than the indium composition, which indicates that as the confinement energy increases within the nanodisk, the ground-state energy becomes large enough such that the piezoelectric field, strain, and bandgap starts to have less influence on the absorption characteristics.

**Figure 4:**
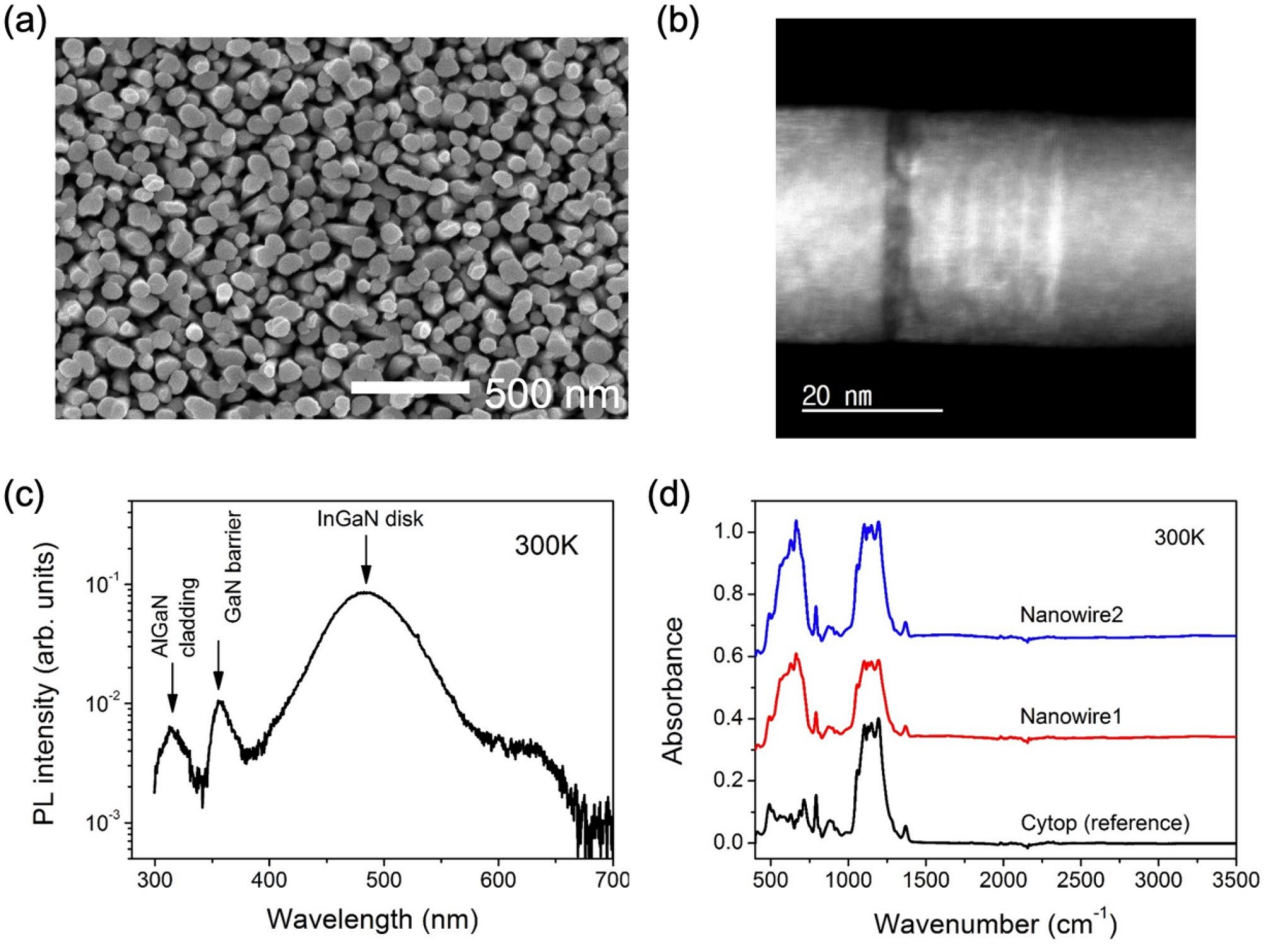
(**a**) Top-view SEM and (**b**) TEM image of the nanowire sample. (**c**) Photoluminance intensity of the as-grown nanowire sample measured with a continuous wave laser emitting at 266 nm, (**d**) intraband absorption spectra of the nanowires embedded in CYTOP measured by FTIR at room temperature with light incident normal to the sample.

Based on the confirmed theoretical calculation, the InGaN/GaN disk-in-nanowire structures surrounded by AlGaN shell were grown on SiO_2_/Si substrate by using Veeco Gen II MBE system equipped with a radio frequency plasma-assisted nitrogen source (see “Methods” section for further details). The nanowires were inspected for structural integrity and consistency with our simulated structure using a scanning electron microscope (SEM) and a transmission electron microscope (TEM). A top-view SEM image of our nanowire sample is shown in Fig. 4a, showing uniformly distributed and well-aligned nanowires grown on SiO_2_ in the c-plane direction. The diameters of the grown nanowires that include the InGaN/GaN cores and the AlGaN outer shells are varied with a range from 30 to 90 nm. This variation could be originated from the geometry of the growth system where different effective fluxes can be incident on each nanowire during the AlGaN shell growth on the InGaN/GaN nanowire cores^43^. On the other hand, to investigate the nanodisks surrounded by the AlGaN shell, we show a cross-section of the nanowire using the TEM technique. The TEM image (Fig. 4b) clearly shows 2 nm thick InGaN disks sandwiched between 2 nm GaN barriers with 5 nm thick AlGaN surrounding the core, with sharp heterointerface. We did not observe any extended defects such as dislocations within the nanowires.

The photoluminescence (PL) absorption spectrum, excited with a continuous wave 266 nm laser at T = 300 K, of the as-grown nanowires is shown in Fig. 4c. Three distinct peaks are measured, corresponding to the InGaN nanodisk, GaN barrier, and AlGaN cladding at 480, 356, and 310 nm, respectively. The slight blue-shift of the GaN PL peak is attributed to the compressive strain present in the barrier. Figure 4d shows the absorption spectrum of the semi-freestanding nanowire specimens, using a Fourier transform infrared spectroscopy (FTIR), transferred to a thin CYTOP film with the Si substrate removed (see “Methods” section), along with a control sample consisting of just the CYTOP film at room temperature with normal incident light. An absorption peak at 82 meV, corresponding to a wavelength of approximately 15 μm, is observed for samples with the nanowires, and absent from the control sample. The absorption peak had a linewidth of about 20 meV due to a slight size variation of the nanodisks. These results demonstrate absorption of TE polarized light perpendicular to the growth direction, which is difficult to achieve using a planar quantum well due to the selection rule. For planar quantum wells, light must be incident at an angle, or an additional surface patterning is needed for intraband absorption, which increases the complexity of operation and fabrication.

## Conclusion

In summary, we demonstrate mid-IR intraband absorption of normal incident TE polarized photons in InGaN/GaN nanodisks cladded with AlGaN. Theoretically, we confirm that the large piezoelectric field at the interface of the InGaN/GaN as well as the strain compensating AlGaN shell creates a highly confined well within the InGaN nanodisk. The momentum matrix of the intraband transitions show that transitions from the ground state into degenerate *p*_*x*_, *p*_*y*_ orbital-like excited states allow absorption of normal incident TE and TM polarized light. Experimentally, FTIR measurements confirm the intraband absorption at a wavelength of 15 µm, corresponding to a transition energy of 82 meV. Absorption energies as a function of indium composition and nanowire diameter indicate possibility of fine tuning the absorption energies in the IR spectrum, and demonstrates the versatility of nanodisk-in-nanowire structures for electro-optical applications.

## Methods

### Epitaxy growth via MBE system

The InGaN/GaN disk-in-nanowire structures surrounded by AlGaN shell were grown on SiO_2_/Si substrate by using Veeco Gen II MBE system equipped with a radio frequency plasma-assisted nitrogen source. GaN:Ge and GaN:Mg epitaxial layers were grown at a substrate temperature of 780 °C, nitrogen flow rate of 1.0 sccm, forward plasma power of 350 W, and a Ga beam equivalent pressure (BEP) of 8.1 × 10^−8^ Torr. During the growth of the active region, the substrate temperature was reduced to 690 °C to enhance indium incorporation in the quantum disks. Lastly, AlGaN shell layer was grown at the substrate temperature of 780 °C with Al BEP of 1.2 × 10^−8^ Torr and Ga BEP of 5.1 × 10^−8^ Torr.

### Sample preparation

To measure the absorption rate, the nanowire is transferred to a CYTOP (CTL 809 M) membrane. First a thin layer of CYTOP is spread on the top surface of the nanowire sample. After baking at 50 °C, 80 °C, and 250 °C for 20 min, 30 min and 45 min respectively, the sample is dipped in 100% HF solution for SiO_2_ etching. The Si substrate can be removed once the SiO_2_ is fully etched, leaving the nanowire and CYTOP only, as shown schematically in Fig. S1.

## Supplementary Information

Supplementary Information.

## Data Availability

The datasets generated during and/or analyzed during the current study are available from the corresponding author on reasonable request.
