# Supplementary material for: Mid-infrared photon sensing using InGaN/GaN nanodisks via intersubband absorption
Source: Sci Rep. 2022 Mar 11;12:4301. doi: 10.1038/s41598-022-08323-9 (PMC8917152; doi:10.1038/s41598-022-08323-9)
Supplement: Supplementary file 1 — Supplementary Information. [file 41598_2022_8323_MOESM1_ESM.docx]

**Supplementary Information**

**Mid-Infrared Photon Sensing using InGaN/GaN Nanodisks via Intersubband Absorption**

Zhang Xing^1,§^, Afroja Akter,^2,§^ Hyun S. Kum,^3,§^ Yongmin Baek,^4,§^ Yong-Ho Ra,^5^ Geonwook Yoo,^6^ Kyusang Lee,^4^ Zetian Mi,^7^ and Junseok Heo^2,*^

*^1^ Semiconductor industry and technology research Institute, Jimei University, Xiamen 361021, China*

*^2^Department of Electrical and Computer Engineering, Ajou University, Suwon 16499,
South Korea*

*^3^Department of Electrical and Electronic Engineering, Yonsei University, Seoul 03722, South Korea*

*^4^Department of Electrical and Computer Engineering, University of Virginia, Charlottesville, Virginia 22904, USA*

*^5^Optic & Electronic Component Material Center, Korea Institute of Ceramic Engineering & Technology, Jinju 52851, South Korea*

*^6^School of Electronic Engineering, Soongsil University, Seoul 06978, South Korea*

*^7^Department of Electrical Engineering and Computer Science, University of Michigan, Ann Arbor, Michigan 48109, USA*

^*^Corresponding author: jsheo@ajou.ac.kr

**1. Strain Calculation**

After experiencing a uniform deformation, the axes of the heterostructure are distorted from *x*, *y*, *z* to , ,

The strain calculation is based on a continuum mechanical model based on classical elasticity. The components of the strain tensor in terms of the displacement vector can be written as follows [1].


$$\varepsilon_{ij}\left( x \right)=\frac{1}{2}\left( \frac{\partial u_{i}(x)}{\partial x_{j}}+\frac{\partial u_{j}(x)}{\partial x_{i}} \right) (1.1)$$

where and run over x, y and z. The vector describes the displacement due to lattice deformations. Diagonal components of the strain tensor measure the extensions per unit length along the coordinate axes (positive values mean tensile strain, negative values compressive strain) and off-diagonal elements measure shear deformations. The diagonal matrix must not necessarily coincide with the crystal or simulation coordinate system. The strain tensor components can be determined by minimizing the elastic energy *E*,


$$E=\frac{1}{2}\int\left( \sum_{i}^{6} \sum_{j}^{6} C_{ij}\left[ \varepsilon_{i}\left( x \right)-\varepsilon_{i}^{0}(x) \right]\left[ \varepsilon_{j}\left( x \right)-\varepsilon_{j}^{0}(x) \right] \right)dv (1.2)$$

where are the elastic strain components, and are the local intrinsic strain components which come from the lattice mismatch between the interfaces of the semiconductor materials in the heterostructure. are the elastic stiffness components. Since our nanowire is based on a hexagonal wurtzite material, we can consider five elastic stiffness components, namely, , , ,, and local intrinsic strain components can be found as follows, [2] and.

, and are the lattice constant of In_0.3_Ga_0.7_N, GaN and Al_x_Ga_1-x_N respectively. The correspondence between Eq. A.1 and Eq. A.2 are,, , , and .

**2.** **Intraband Momentum Matrix and Absorption Calculation for the Nanowire Structure**

Assuming Fermi- Dirac distribution (completely filled) and (empty), from Fermi’s golden rule the transition rate for the absorption of a photon is as in Eq. 2.1, [2-3]


$$W_{abs}=\frac{2\pi}{\hbar}\left| \left\langle b | H^{'}\left( r \right) | a \right\rangle\right|^{2}\sigma(E_{b}-E_{a}-h\omega) (2.1)$$

Where ‘*a*’ is initial electronic state and ‘*b*’ is the finial electronic state, assuming > . The perturbation Hamiltonian will be,


$$H^{'}\left( r \right)=-\frac{qA_{0}}{2m_{0}}e^{i\vec{\cdot k}_{op}\cdot\vec{r}}\hat{e}\cdot\bar{P}_{ba} \left( 2.2 \right)$$

Here is the momentum matrix element for the intraband transition.

According to the momentum conservation,

,

Where is the crystal momentum of (electron) at the final state and is the crystal momentum of at initial state and is the photon momentum.

, is the lattice constant and <<, we can ignore.

Hence, as $e^{i\vec{\cdot k}_{op}\cdot\vec{r}}=1$,

$$H^{'}\left( r \right)=-\frac{qA_{0}}{2m_{0}}\hat{e}\cdot\bar{P}_{ba} \left( 2.3 \right)$$

$$\bar{P}_{ba}=\left\langle\psi_{b} | \bar{P} | \psi_{a} \right\rangle=\int_{r} \psi_{b}^{*}(\frac{\hbar}{i})\nabla\cdot\psi_{a}d^{3}r (2.4)$$

Here is the complex conjugate of the final electronic state and is the initial electronic state.

The intraband absorption, including broadening effect is [3].

$$\alpha_{a\to b}(E_{b}-E_{a})=\frac{\pi q^{2}}{n_{r}c\varepsilon_{0}\omega m_{0}^{2}}\frac{2}{V}\left| \hat{e}\cdot\bar{P}_{ba} \right|^{2}\times\frac{\gamma/\pi}{{(E_{b}-E_{a}-\hbar\omega)}^{2}+\gamma^{2}} (2.5)$$

where is the refractive index of In_0.3_Ga_0.7_N [4], is speed of light in vacuum, is the total volume of the disk, is the momentum matrix element of ground state to higher excited states, and is the full width at half maximum (FWHM). We assume the FWHM is around 4 meV.

**3. Sample preparation**

To measure the absorption rate, the nanowire is transferred to a cytop (CTL 809 M) membrane. First a thin layer of cytop is spread on the top surface of the nanowire sample. After baking at 50^°^ C, 80^°^ C, and 250^°^ C for 20 minutes, 30 minutes and 45 minutes respectively, the sample is dipped in 100% HF solution for SiO_2_ etching. The Si substrate can be removed once the SiO2 is fully etched, leaving the nanowire and cytop only, as shown schematically in Fig. S1.


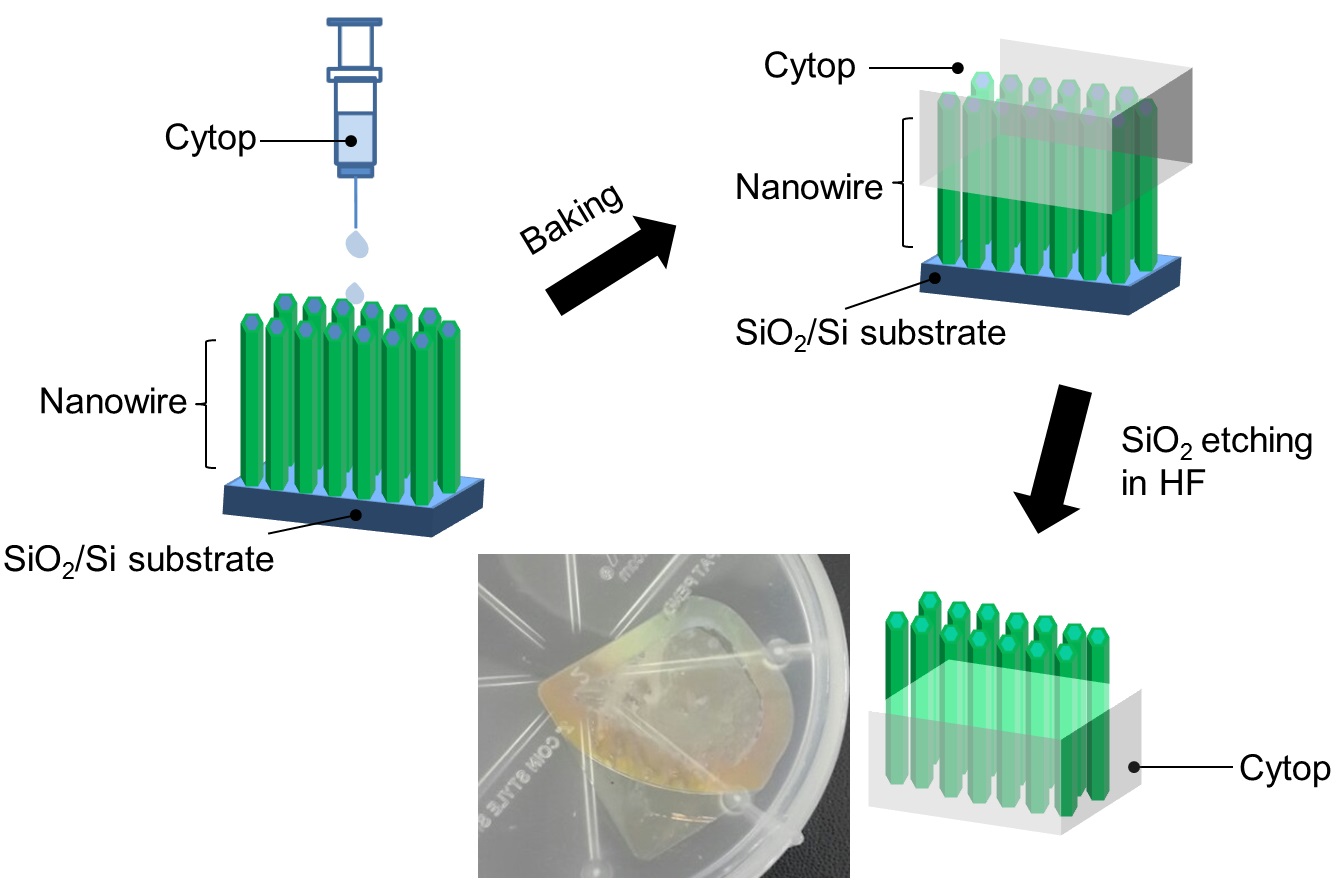


**Fig. S1.** Sample preparation

**4. Fourier transform infrared spectroscopy** **(FTIR)**

The intraband absorption probed by Fourier transform infrared spectroscopy (FTIR) is performed on a Thermo NICOLET iS50 spectrometer using a high stable and long lifetime mid-Far IR (9600 ^~^ 20 cm^-1^) source, a Ge-on-KBr beamspiller (7800 ^~^ 350 cm^-1^), and a DLaTGS w/KBr detector (12500 ^~^ 350 cm^-1^). An electromagnetic drive 90 degree Michelson type interferometer has been employed. The transmission is measured at room temperature in attenuated total reflectance (ATR) mode (where pressure is around 60 psi).

**REFERENCES**

1. C. Rivera, U. Jahn, T. Flissikowski, J. L. Pau, E. Munoz, and H. T. Grahn , Phys. Rev. B 75, 045316 (2007).
2. J. Singh, Electronic and Optoelectronic properties of Semiconductor structures*,* Cambridge University Press, pp. 358-375 (2003).
3. S. L. Chuang , Physics of Photonic Devices*,* 2nd Edition , Wiley, pp. 348-366 (2009).
4. M. Anani, H. Abid , Z. Chamab , C. Mathieu , A. Sayede , B. Khelifa, Microelectronics J. 38, 262-266 (2007).
